# California Fever

**Published:** 1849-07

**Authors:** 


### CALIFORNIA FEVER.

We are sorry to announce that our worthy colleague, Dr. Brown, of St. Louis, has been taken off by this very prevalent disease. We understand he *departed* about the 10th of April, not this life, however; but for the *Sacramento diggings.* We hope it may be a long, long time before his grave may be dug in that region. We believe it is generally known that the Doctor is fond of Mineralogical as well as Geological study; and are informed his removal to California is more for the purpose of extending his researches in this department of science, than for the accumulation of gold.

We are glad to find that among those of our subscribers who have, taken, or are about to take up their abode in this distant part of our country, that instead of discontinuing the Register, they send in their subscription payment in advance, and order it sent to them. Among these is Dr. Stratton, of St. Louis, who accompanies Dr. Brown.

With such emigrants, California must become the most flourishing part of the United States, and Dental Science there keep pace with the improvements of the age.—[CIN. ED.

